# Synthesis of Si, N co-Doped Nano-Sized TiO_2_ with High Thermal Stability and Photocatalytic Activity by Mechanochemical Method

**DOI:** 10.3390/nano8050294

**Published:** 2018-05-02

**Authors:** Peisan Wang, Chunxia Qi, Pengchao Wen, Luyuan Hao, Xin Xu, Simeon Agathopoulos

**Affiliations:** 1 Chinese Academy of Science, Key Laboratory of Materials for Energy Conversion, Department of Materials Science and Engineering, University of Science and Technology of China, Hefei 230032, China; wpsan@mail.ustc.edu.cn (P.W.); wpc39@mail.ustc.edu.cn (P.W.); hly@ustc.edu.cn (L.H.); 2 Department of Chemistry, Anhui Medical University, Hefei 230032, China; 3 Department of Chemical Engineering, Hefei Normal University, Hefei Lianhua Rd 1688, Hefei 230601, China; c200602003@ahu.edu.cn; 4 Department of Materials Science and Engineering, University of Ioannina, GR-451 10 Ioannina, Greece; sagat@cc.uoi.gr

**Keywords:** Si, N co-doped anatase, high-energy mechanical milling, mechanochemical method, photocatalytic activity

## Abstract

Τhe photocatalytic activity in the range of visible light wavelengths and the thermal stability of the structure were significantly enhanced in Si, N co-doped nano-sized TiO_2_, and synthesized through high-energy mechanical milling of TiO_2_ and SiO_2_ powders, which was followed by calcination at 600 °C in an ammonia atmosphere. High-energy mechanical milling had a pronounced effect on the mixing and the reaction between the starting powders and greatly favored the transformation of the resultant powder mixture into an amorphous phase that contained a large number of evenly-dispersed nanocrystalline TiO_2_ particles as anatase seeds. The experimental results suggest that the elements were homogeneously dispersed at an atomic level in this amorphous phase. After calcination, most of the amorphous phase was crystallized, which resulted in a unique nano-sized crystalline-core/disordered-shell morphology. This novel experimental process is simple, template-free, and provides features of high reproducibility in large-scale industrial production.

## 1. Introduction

The strong and increasing presence of organic contaminants currently in the environment leads to many studies about photocatalytic degradation of photocatalytic materials. Titanium dioxide (TiO_2_) is a very popular material in such applications and it owes its existence to its non-toxicity, high chemical stability, low cost, and good optoelectronic properties [1,2,3,4,5]. Anatase, which is one of the mineral forms of TiO_2_, is more active under ultraviolet (UV) light irradiation than the other TiO_2_ crystalline phases such as brookite and rutile [6,7,8]. Anatase TiO_2_ has a 3.2 eV wide band gap. Nevertheless, due to this value, only an approximate 3% of the arriving solar energy on earth can be used by anatase TiO_2_ [9,10,11].

Anatase TiO_2_ is thermodynamically a high-temperature metastable structure, which can irreversibly transform into rutile TiO_2_ after calcination at ca. 600 °C. Although rutile has a smaller band gap (=3.0 eV) than anatase, it exhibits poor photocatalytic activity because of the intrinsic features of the crystaline structure of rutile [12,13,14]. Furthermore, anatase TiO_2_ synthesized at low temperatures has poor crystallinity, which deteriorates its photocatalytic activity [15].

For the sake of substantially improving the solar energy as well as the thermal stability of anatase TiO_2_, many studies have reported on the development of photocatalysts, which display high activity by irradiation with ultraviolet light as well as in the range of visible light [16,17,18,19]. The visible-light response of N-doped TiO_2_ was covered initially in 1986 by Sato [20]. Since then, many research groups have reported on the visible-light response of N-doped TiO_2_ materials produced by various approaches. Nonetheless, N is not stable in TiO_2_ at high temperatures since it can be decomposed at about 600 °C [21]. In our earlier study, anatase TiO_2_ was also doped with Si, which resulted in a stable anatase phase at high calcination temperatures. The high thermal stability of N in anatase TiO_2_ was attributed to the existence of Si-N bonds in TiO_2_ [22]. It was also demonstrated that the homogeneous dispersion of Si in the TiO_2_ crystal plays an important role in photocatalytic activity.

High-energy mechanical milling is often utilized to synthesize various non-equilibrium and equilibrium alloy or ceramic phases [23,24,25]. This solid-state powder processing includes repeated fracture, welding, and re-welding of powder particles. Therefore, it can produce highly homogeneous precursors and equally homogeneous resultant powders. Currently, high-energy mechanical milling is also considered an effective process for synthesizing a variety of non-equilibrium nano-structured phases [26,27].

This paper proposes the high-energy ball-mill solid-state reaction method for synthesizing Si, N co-doped nano-sized anatase TiO_2_ because it is a simple and template-free process. It ensures high reproducibility and exhibits a great potential for large-scale industrial production.

## 2. Materials and Methods

Fine powders of anatase TiO_2_, rutile TiO_2_ and nano-SiO_2_ were used as raw materials (Aladdin Chemical Co., Ltd., Shanghai, China). First, powders of TiO_2_ doped with Si were prepared. These samples had various weight ratios of Ti/Si, which were 0.999:0.001, 0.995:0.005, 0.99:0.01, 0.98:0.02, and 0.96:0.04. Hereafter, these samples are simply denoted as 0.1Si, 0.5Si, 1Si, 2Si, and 4Si, respectively. The mixtures of TiO_2_ and SiO_2_ powders were ground in an agate mortar by hand for 20 min. This manually-prepared mixture was transferred to a silicon nitride ball-milling tank of a high-energy ball mill. The milling speed was set as 800 rpm and the milling time as 6 h.

A non-doped (i.e., with no Si) sample of pure anatase TiO_2_, denoted as 0Si, was also prepared under the same conditions of high-energy ball milling.

In order to investigate the influence of the speed of ball-milling, the sample 2Si was subjected to ball-milling at various speeds. More specifically 500 rpm, 600 rpm, and 800 rpm and these samples are denoted as H-500, H-600, and H-800, respectively.

To produce the Si, N co-doped TiO_2_ samples, the obtained Si-doped TiO_2_ powders (0Si–4Si) were transferred to Al_2_O_3_ crucibles and then calcined at 600 °C for 4 h in an atmosphere of a flowing gas mixture of ammonia (50%)/N_2_ (50%) in a high-temperature tubular electric furnace. The heating rate was set as 4 K/min. After the thermal treatment, the samples were cooled down to room temperature naturally. These Si, N co-doped samples are designated as 0Si-N, 0.1Si-N, 0.5Si-N, 1Si-N, 2Si-N, and 4Si-N.

Two samples with a Ti/Si ratio of 0.98:0.02 were produced similarly using rutile TiO_2_ and they are denoted as R-2Si, R-2SiN.

The identification of the crystalline phases was carried out by XRD (X-ray diffraction analysis, PW 1700, Philips Research Laboratories, Eindhoven, The Netherlands) using Cu K_α1_ radiation and α scanning rate of 2 °C/min. The nano-structure of the samples was observed using an HRTEM (high-resolution transmission electron microscope, JEOL-2010, JEOL, Tokyo, Japan). An EDS (energy dispersive X-ray spectrometer), FTIR (Fourier transform infrared spectra, Nicolet 8700, Thermo Scientific, Waltham, MA, USA), and XPS (X-ray photoelectron spectra, ESCALAB 250, Thermo-VG Scientific, USA) were used for elemental and chemical bond analysis. Thermal analysis of the samples was conducted (DTA-TGA, DTG-60H, Shimadzu, Kyoto, Japan) at a heating rate of 10 K/min up to 1200 °C. The specific pore volume and surface area were determined with Barrett-Joyner-Halenda (BJH) and Brunauer-Emmett-Teller (BET) reanalysis by using Micromeritics Tristar II 3020M equipment (Micromeritics, Norcross, GA, USA). The photoluminescence properties of the produced samples were measured by using the fluorescent spectrophotometer (PL, F-4600, Hitachi Ltd., Tokyo, Japan) with a 200 W Xe-lamp as an excitation source at room temperature. The UV-visible spectrophotometer (SOLID 3700, Shimadzu Ltd., Kyoto, Japan) was used to measure UV-visible diffuse reflectance spectra of the produced samples.

The photocatalytic activity was surveyed by the decomposition of RhB (Rhodamine B). In a representative experiment, the 50 mg synthesized catalyst powder were added to a 50 mL RhB aqueous solution with a concentration of 10 mg/L in a clean quartz reactor. The suspension liquid was magnetically stirred with uniform speed in a dark room for 30 min before using illumination to establish adsorption/desorption equilibrium between the surface of the photocatalyst and RhB. After that, an Xe lamp of 500 W was used as a UV-visible light source. The 5 mL mixture was centrifuged with 8000 rpm rotational speed to remove the catalyst particles and take them out in fixed illumination time intervals. The RhB concentration was determined by a UV-visible spectrophotometer (UV-759, Shanghai Precision & Scientific Instrument Co., Ltd., Shanghai, China). For comparison, the photocatalytic activity of a commercial photo catalyst Degussa P25 TiO_2_ (Evonik Degussa, Essen, Germany), raw anatase, and raw rutile were also measured under the same condition.

## 3. Results and Discussion

### 3.1. Crystalline Structure, Nanostructure, and Elemental Analysis

**Figure 1:**
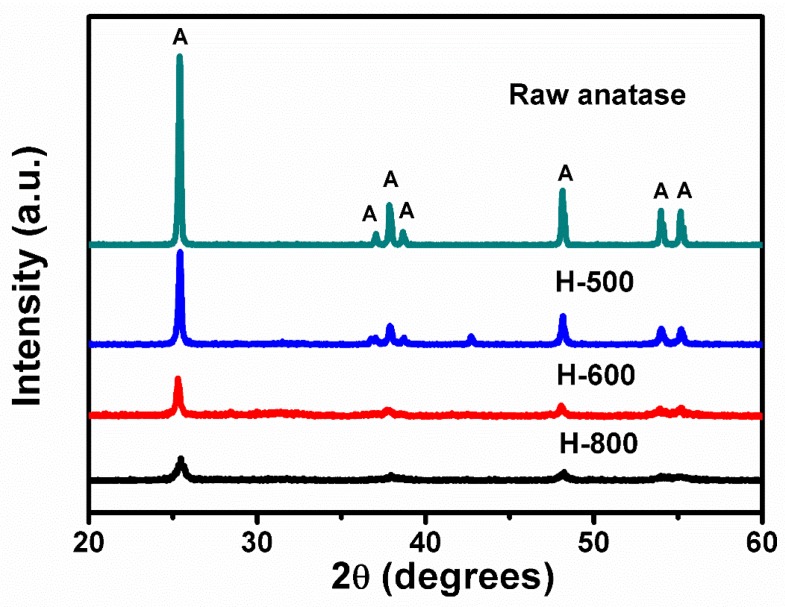
Influence of ball-milling speed on the crystalline structure (A: anatase TiO_2_, JCPDS#21-1272).

The diffractograms in Figure 1 suggest that the speed of ball-milling has a significant impact on the crystalline structure of the (non-calcined) produced samples. More specifically, the diffraction peaks of the anatase phase become markedly weaker and broader when the speed of the high-energy ball-milling increases. This is clearly observed in the peak of the plane (101) at ~25.3°. The reduction in the intensity of the peaks suggests that the highly crystallized anatase phase gradually shifts to a regime of an amorphous state along with a considerable refinement in the size of the particles. The average dimension of the crystallites (D), calculated by the Scherrer equation [28,29], D = Kλ/Bcosθ, where K is a constant near 1, λ is the wavelength of the X-ray radiation, B is the FWHM (full width at half maximum), and θ is the angle of diffraction, were 73.54 nm, 42.12 nm, 27.39 nm, and 17.01 nm for the samples of TiO_2_ raw anatase, H500, H600, and H800, respectively.

**Figure 2:**
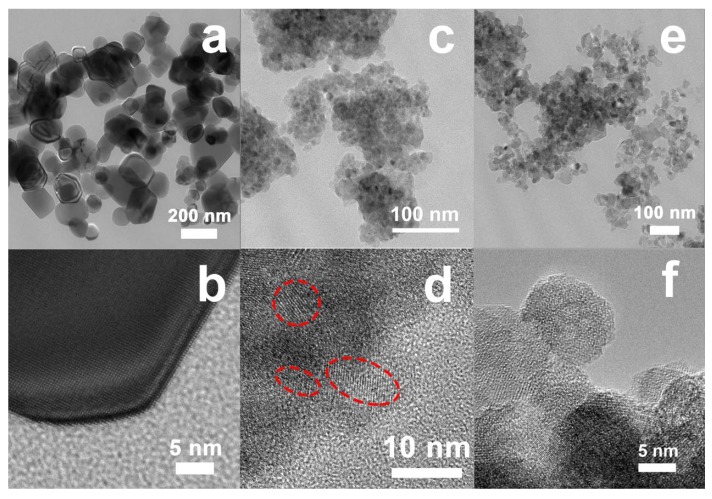
Transmission electron microscope (TEM) (**a**,**c**,**e**) and high-resolution transmission electron microscope (HRTEM) (**b**,**d**,**f**) images of the powders of the raw anatase TiO_2_ (**a**,**b**) and the samples 2Si (**c**,**d**) and 2Si-N (**e**,**f**) milled at the speed of 800 rpm.

These results are in broad agreement with the images of TEM and HRTEM (see Figure 2a–d). The powder of raw anatase TiO_2_ is well crystallized and the particle size ranges between 50 nm and 200 nm (see Figure 2a,b). High-energy ball-milling at 800 rpm resulted in a significant decrease in particle size in the sample 2Si down to about 5~10 nm, which was observed in the areas included inside the dashed lines in Figure 2d and features of amorphous material are also observed (see Figure 2c,d).

The above results suggest that the mechano-chemical processing via the high-energy ball-milling causes violent collision, grinding, and an abrasive effect on the powder, which resulted in grain refinement and eventually destroyed the original surface of the powder as far as its morphology and chemistry are concerned. Therefore, the surface lattice of the TiO_2_ crystal is transformed to an amorphous layer and the crystalline regime decays.

**Figure 3:**
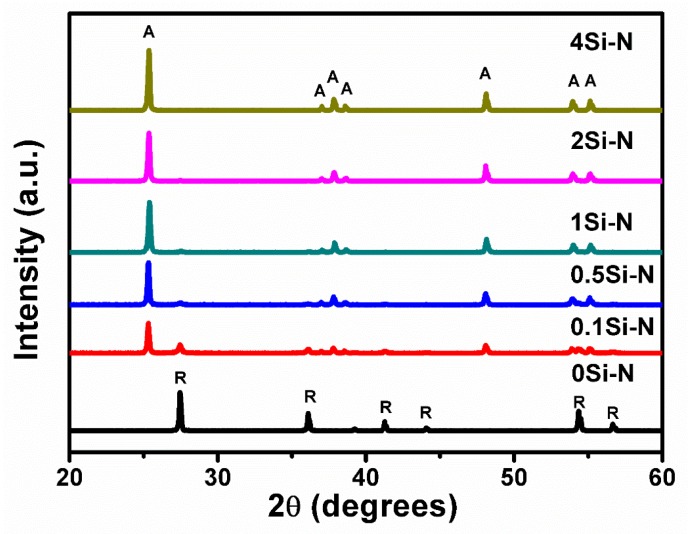
Influence of Si-content on the crystalline structure of the Si, N co-doped samples and the Si-free sample 0Si-N (A: anatase TiO_2_, JCPDS#21-1272, R: rutile TiO_2_, JCPDS#21-1276).

The diffractograms in Figure 3, which were obtained from the Si-free sample (0Si-N) and Si, N co-doped samples 0.1Si-N–4Si-N calcined at 600 °C and demonstrate the essential role of Si in the formation of anatase phase in the final product. More specifically, the rutile phase was solely formed in the sample 0Si-N while pure anatase was developed in the 2Si-N and 4Si-N powders. Anatase was predominantly formed in the samples 0.1Si-N–1Si-N, but small peaks attributed to traces of the rutile phase were also recorded in these samples.

**Figure 4:**
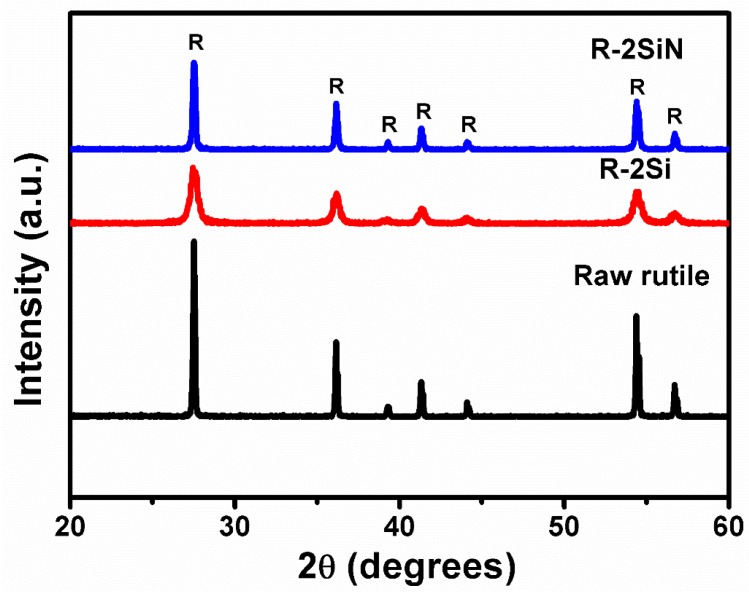
X-ray diffractograms of the rutile-containing samples.

Nonetheless, the X-ray diffractograms of Figure 4 suggest that the presence of anatase seeds is necessary in order to get anatase. To be more specific, these diffractograms show that in the rutile-containing samples R-2Si and R-2SiN, rutile is exclusively formed regardless of the fact that they both contained Si. However, high-energy ball-milling also caused reduction in the crystallinity in these samples, which was suggested by the widening of the diffraction peaks while calcination improved crystallinity (i.e., the peaks become sharper from R-2Si to R-2SiN with the latter being calcined at 600 °C).

The TEM and HRTEM images of the sample 2Si-N, shown in Figure 2e,f, respectively, suggest that this powder (2Si-N) is uniform (i.e., it exhibits a narrow particle size distribution) with a particle size of about 40 nm to 50 nm. Accordingly, mechanical milling should favor diffusion and atomic rearrangements of the starting powders. The homogeneous amorphous mixture should eventually lead to formation of anatase phase where Si should be uniformly distributed. As a result, a unique nano-sized crystalline-core/disordered-shell morphology is observed.

**Figure 5:**
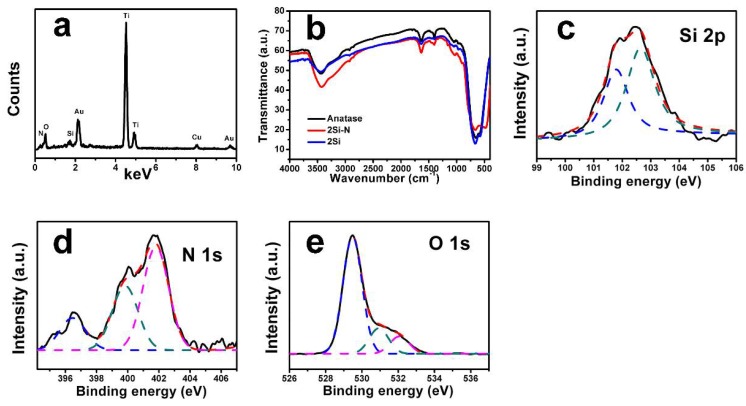
Results of (**a**) energy dispersive X-ray spectrometer (EDS) (the peaks at 2.1 keV and 9.7 keV are due to the conductive Au coating), (**b**) Fourier transform infrared spectra (FTIR), and (**c**,**d**,**e**) X-ray photoelectron spectra (XPS) analyses of the sample 2Si-N.

The sample 2Si-N was thoroughly analyzed by EDS, FTIR, and XPS (see Figure 5). The presence of TiO_2_ is indicated by the peaks at 0.5 keV for O and 4.5 keV and 4.9 keV for Ti (EDS, Figure 5a). The presence of N and Si is confirmed by the peaks of N (0.4 keV) and Si (1.7 keV). Nitrogen contents determined by the XPS were 0.09%, 0.15%, 2.15%, 3.21%, and 4.33% for the sample nSi-N (n = 0.1, 0.5, 1, 2, and 4), respectively.

In the FTIR spectra of raw anatase and the samples 2Si and 2Si-N (see Figure 5b), a broad band at 2700–3600 cm^−1^ attributed to O-H stretching vibrations of water, a peak at 1635 cm^−1^ was ascribed to the flexion vibrations of the O-H groups [30,31]. A broad band at 400–800 cm^−1^, which is owed to the stretching vibrations of O-Ti-O and Ti-O bonds [32,33], was recorded. In the sample 2Si-N, the absorption peak of Si-N bond, situated at 890 cm^−1^, was also observed [34].

The presence of Si-N and Si-O bonds was also confirmed by the XPS analysis. More specifically, the two peaks at 102.7 eV and 101.7 eV correspond to the Si-N and Si-O bonds, respectively (see Figure 5c) [35,36]. Three peaks are discovered in the spectrum of N 1s (see Figure 5d). The peaks at 399.9 eV and 401.8 eV are ascribed to Ti-O-N or Ti-N-O, respectively [37,38,39]. The peak at 396.5 eV is due to negatively-charged nitrogen that substitutes oxygen and forms Si-N and Ti-N bonds in the lattice of TiO_2_ [39]. An O 1s core level peak emerges at ca. 529.5 eV, 530.9 eV, and 532.1 eV (see Figure 5e) [35,36,40]. The peak at 529.5 eV is the main peak, which can be ascribed to the oxygen in Ti-O-Ti. The peak at 530.9 eV is attributed to Ti-O-Si linkage and the peak at 532.1 eV is attributed to the O-H band on account of the water adsorption.

**Figure 6:**
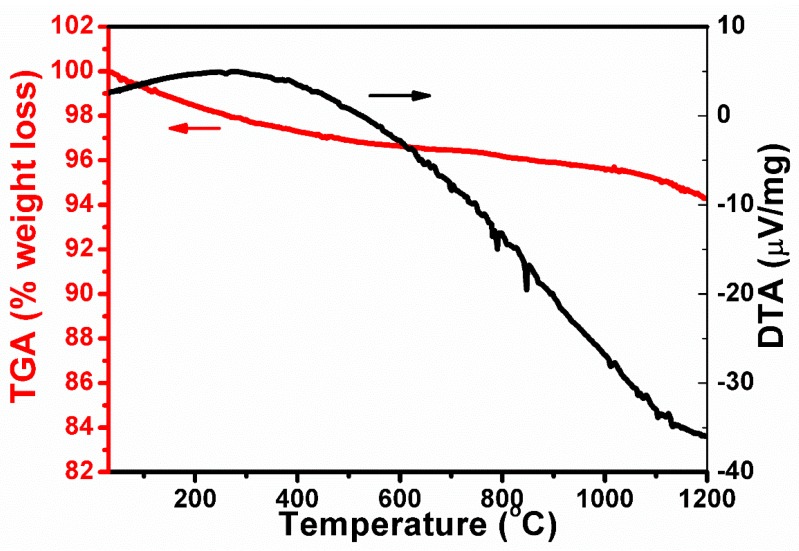
Results of thermal analysis (DTA and TGA) of the sample 2Si-N.

The thermal analysis of the sample 2Si-N (see Figure 6) showed a slight weight loss below 600 °C. This occurred because of the evaporation of the adsorbed, crystallized water as well as the decomposition of the ammonium groups in the sample. The two weak endothermic peaks close to 800 °C and a small weight loss in the range 800 °C to 1000 °C might be attributed to the decomposition/release of internal N. The weight loss at temperatures higher than 1000 °C might be ascribed to a result coming from the breaking of Si-N bonds. The results are in satisfactory agreement with our previous reports [22], which confirms the stability of the obtained sample.

### 3.2. Textural Parameters

In this section, the key features, which are closely related to the catalytic efficiency of the produced photocatalysts are presented using namely texture, photoluminescence properties (emission spectra and UV-visible diffuse spectra), and photocatalytic activity, which were experimentally determined by the degradation of RhB.

**Figure 7:**
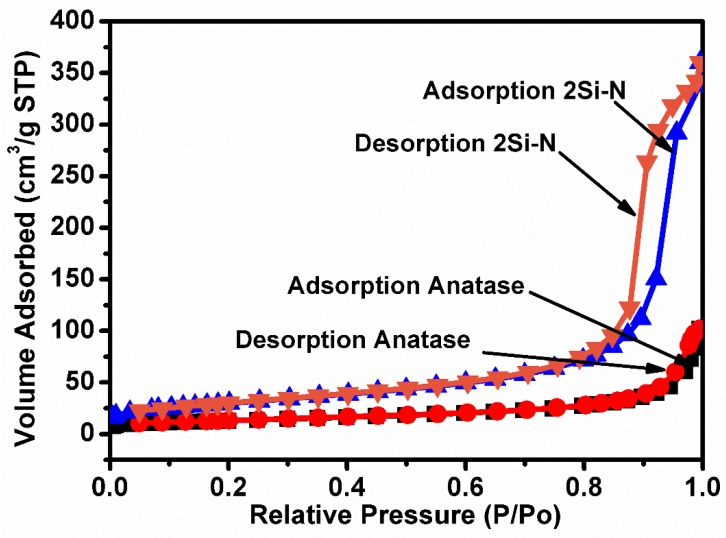
N_2_ adsorption/desorption isotherms of the samples 2Si-N and raw anatase TiO_2_ powder.

Texture, associated with specific surface area and pore size distribution, is an important feature of a catalyst. The N_2_ adsorption-desorption curves of the samples 2Si-N and raw anatase TiO_2_ powder were experimentally recorded and the results are plotted in Figure 7. It is immediately concluded that the raw anatase TiO_2_ powder is a well-crystallized and a non-porous material because of the absence of the hysteresis loop. On the contrary, the hysteresis loop between the adsorption and desorption isotherms in the sample 2Si-N suggests that a microporous structure was developed as a result of the high-energy ball-milling treatment, which led to an increase in the specific surface area from 45.40 m^2^/g for the raw TiO_2_ to 107.69 m^2^/g in the sample 2Si-N.

### 3.3. Optical Properties

The optical properties of the solid material are often correlated to the microstructure with regard to its influence on the electron state, the defect state, and energy levels [41,42]. In the particular case of nano-structured materials, the photoluminescence spectra are closely related to the migration of photo-generated electrons and holes as well as their separation and recombination rate.

**Figure 8:**
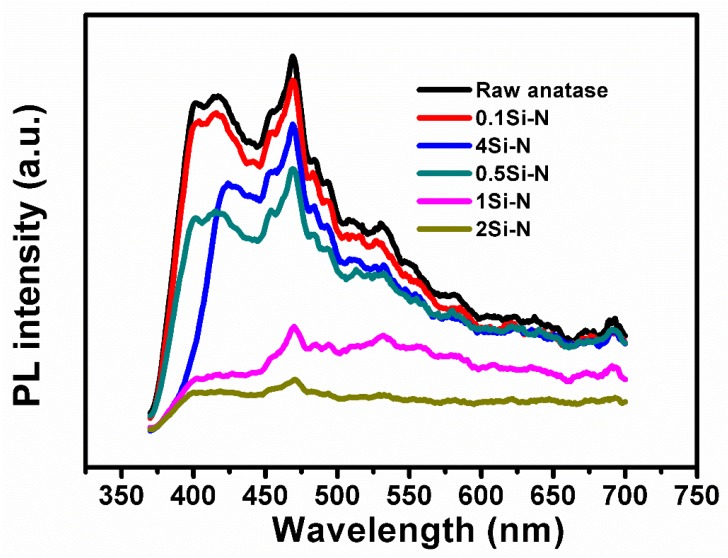
Photoluminescence properties (emission spectra) of the produced samples and comparison with raw anatase TiO_2_ powder.

The photoluminescence properties of the produced samples are reported in the emission spectra of Figure 8. The emission spectrum of raw anatase powder was also recorded for comparison purposes. Two peaks were recorded at 410 nm and 470 nm. The former (410 nm) should correspond to the forbidden band width of the anatase phase TiO_2_ [43]. The latter (470 nm) should be mainly due to oxygen vacancies or defects on the surface of TiO_2_. The highest emission intensity was recorded in the raw anatase powder. Additionally, a small amount of Si (0.1Si-N) immediately caused a decrease in the intensity. The lowest intensity was recorded in the sample 2Si-N.

These results suggest that Si doping should cause a certain defect to the TiO_2_ lattice, which acts as an electron-capturing agent. The theory suggests that the utilization of photo-generated electrons and holes increases when the photo-generated electrons and holes are effectively separated. Accordingly, the decrease in fluorescence intensity from the 0.1Si-N sample to the 2Si-N sample should mean that the photo-generated electron-hole separation is suppressed with the increase in Si content (in this range of Si-content). Nonetheless, a further increase in the Si led to an increase in fluorescence intensity. This might be attributed to formation of many defects in the TiO_2_ lattice, which should act as a recombination center of the photo-generated electron-hole composite. This enhances the fluorescence in 4Si-N.

**Figure 9:**
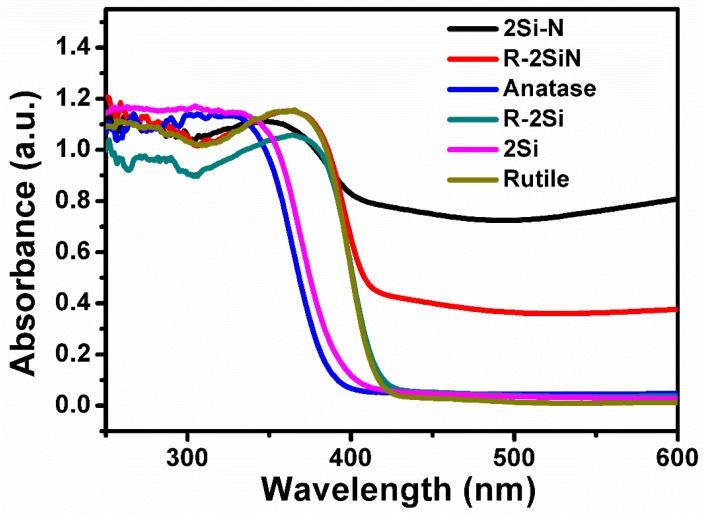
UV-visible diffuse reflectance spectra of the produced samples and comparison with raw anatase and rutile powders.

The UV-visible diffuse reflectance spectrum shows the range of the light response of a material. A good photocatalyst must maximize the utilization of the natural light, which means that it must have a large range of light response (shown in the absorption edge, which is the wavelength threshold of light response) and the absorption intensity of the spectrum. The UV-visible diffuse reflectance spectra of the produced samples and the raw anatase and rutile powders are presented in Figure 9.

First of all, the absorption edge of the pure rutile TiO_2_ is red-shifted when compared to pure anatase TiO_2_ because of the smaller band gap of anatase TiO_2_ (the band gap of rutile TiO_2_ and anatase TiO_2_ are 3.0 eV and 3.2 eV, separately). However, due to the intrinsic structural features of rutile TiO_2_, the photocatalytic performance of rutile TiO_2_ is worse than anatase. Single doping of rutile TiO_2_ with Si via the high-energy ball-milling (which should favor the formation of defects in the sample R-2Si) has a negligible effect on the diffuse reflectance spectra when compared to pure rutile TiO_2_. Co-doping with Si and N in the rutile containing sample R-2SiN only influenced the intensity of absorption in the range of visible light. Accordingly, these results suggest that Si, N co-doping is not expected to improve the photocatalytic properties of rutile TiO_2_.

In the anatase-containing materials, the absorption spectrum of the sample 2Si showed a slight red shift with respect to pure anatase TiO_2_. This can be ascribed to the effect of high-energy ball-milling, which should change the original surface of the TiO_2_ crystal, and to the incorporation of Si atoms into the surface. However, a pronounced red shift of the absorption spectrum was achieved in the Si, N co-doped sample 2Si-N. This is clear evidence of the effective and successful doping of N atoms into the TiO_2_ lattice of anatase phase, which replaced the O atoms. Accordingly, the energy band of anatase TiO_2_ can be suitably adjusted and a larger range of visible light response can be achieved.

### 3.4. Photocatalytic Properties

**Figure 10:**
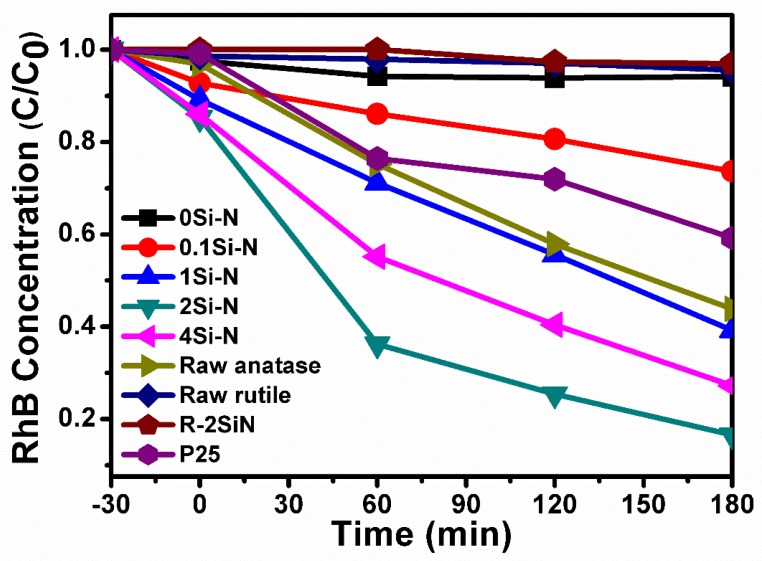
Photocatalytic degradation of RhB using the produced samples and raw anatase and rutile powders.

The plots of Figure 10 actually represent the RhB degradation in an aqueous solution over exposure time in a UV-visible light irradiation by an Xe lamp of 500 W, which is a direct index of the photocatalytic activity of the produced materials in comparison with raw powders of P25, anatase, and rutile TiO_2_. As indicated in the experimental part, the suspension with the photocatalyst was placed in a dark room for 30 min, which established adsorption/desorption equilibrium between the surface of the photocatalyst and RhB and then turned on the illuminant. Therefore, the decline of RhB concentration in this stage between −30 min and 0 min reflects the RhB adsorption behavior. During this step, the results show that the adsorption behavior of RhB on the samples 0Si-N, R-2SiN, and the raw rutile TiO_2_ were very weak. This means that the TiO_2_ rutile phase has low adsorption ability for RhB. On the contrary, the adsorption ability of the anatase-containing samples raw anatase and 0.1Si-N–4Si-N were certainly higher than the rutile phase and it increased with the rise in the content of Si in the anatase phase. In the low Si-content samples 0.1Si-N–1Si-N, which also have small amounts of rutile phase TiO_2_ (see Figure 3), the adsorption ability increased with the rise in the content of anatase phase TiO_2_. The samples 2Si-N and 4Si-N had the higher (i.e., the best) adsorption ability.

Poor photocatalytic activity of the rutile-containing samples 0Si-N, R-2SiN and the raw rutile TiO_2_ was recorded (there is negligible decrease in RhB concentration) while all the anatase-containing samples displayed much better photocatalytic activity over the investigated exposure time. The moderate photocatalytic activity of the sample 0.1Si-N is ascribed to the co-existence of the rutile phase along with the anatase TiO_2_ phase. The photocatalytic property of 1Si-N is better than the raw anatase and P25 because of the co-doped Si and N. The increase in the Si content greatly improved the photocatalytic activity and the best photocatalytic performance was achieved with the sample 2Si-N. Assuming that the increase in Si favors the increase of N incorporation, these results suggest that N incorporation strongly affects the photocatalytic activity of anatase containing powders. N doped TiO_2_ photocatalyst makes a smaller band gap and a wider photo response range than the pure TiO_2_. The photo-generated holes on the surface of the photocatalyst abstracts the electrons of the water molecules in the solution, which can generate many highly oxidizing radicals like hydroxyl radicals (•OH), peroxy radical (O^2−^•), and hydrogen peroxide radicals (•HO^2−^). The highly oxidizing radicals can decompose the organics and finally convert them into water and carbon dioxide.

Nevertheless, the photocatalytic activity decreases slightly in the sample 4Si-N, which is due to an excess of lattice defects. This means that when Si content is too high, many defects are formed in the TiO_2_ lattice, which would act as a recombination center of the photo-generated electron-hole composite and lead to a decrease in the utilization of photo-generated carrier and eventually to a reduction in the photocatalytic activity of the product.

In general, the increase in the photocatalytic activity of the produced Si, N co-doped nano-sized anatase-containing samples can be attributed to the unique crystalline-core/disordered-shell morphology. The synergistic effect owing to the presence of oxygen vacancy’s in the bulk anatase crystalline phase and on the surface, which has nearly stoichiometric disorder features, can effectively narrow the band-gap [44].

## 4. Conclusions

High thermal stability and high photocatalytic activity of Si, N co-doped anatase TiO_2_ phase was achieved by high-energy ball-milling and heat treatment of the produced samples in an ammonia atmosphere. The experimental results showed that rutile TiO_2_, co-doped with Si and N evenly, has a poor photocatalytic activity. On the other hand, doping of anatase TiO_2_ with 2% Si resulted in samples with pure anatase crystalline phase that exhibited the best photocatalytic activity. This high photocatalytic activity is attributed to the Si-N incorporation and the development of a crystalline-core/disordered-shell morphology. This innovative process is simple and provides features of high reproducibility and suitability for large-scale industrial production.
